# Platform dependence of inference on gene-wise and gene-set involvement in human lung development

**DOI:** 10.1186/1471-2105-10-189

**Published:** 2009-06-19

**Authors:** Rose Du, Kelan Tantisira, Vincent Carey, Soumyaroop Bhattacharya, Stephanie Metje, Alvin T Kho, Barbara J Klanderman, Roger Gaedigk, Ross Lazarus, Thomas J Mariani, J Steven Leeder, Scott T Weiss

**Affiliations:** 1 Channing Laboratory, Brigham and Women's Hospital, 181 Longwood Avenue, Boston, MA 02115, USA; 2 Department of Neurosurgery, Brigham and Women's Hospital, Boston, MA 02115, USA; 3 Harvard Medical School, Boston, MA 02115, USA; 4 Center for Genomic Medicine, Brigham and Women's Hospital, Boston, MA 02115, USA; 5 Children's Mercy Hospital, Division of Pediatric Pharmacology and Medical Toxicology, Kansas City, MO 64108, USA; 6 Department of Pediatrics, University of Rochester School of Medicine and Dentistry, Rochester, NY 14642, USA

## Abstract

**Background:**

With the recent development of microarray technologies, the comparability of gene expression data obtained from different platforms poses an important problem. We evaluated two widely used platforms, Affymetrix U133 Plus 2.0 and the Illumina HumanRef-8 v2 Expression Bead Chips, for comparability in a biological system in which changes may be subtle, namely fetal lung tissue as a function of gestational age.

**Results:**

We performed the comparison via sequence-based probe matching between the two platforms. "Significance grouping" was defined as a measure of comparability. Using both expression correlation and significance grouping as measures of comparability, we demonstrated that despite overall cross-platform differences at the single gene level, increased correlation between the two platforms was found in genes with higher expression level, higher probe overlap, and lower p-value. We also demonstrated that biological function as determined via KEGG pathways or GO categories is more consistent across platforms than single gene analysis.

**Conclusion:**

We conclude that while the comparability of the platforms at the single gene level may be increased by increasing sample size, they are highly comparable ontologically even for subtle differences in a relatively small sample size. Biologically relevant inference should therefore be reproducible across laboratories using different platforms.

## Background

The rapid development of microarray technologies has resulted in numerous microarray platforms that are analyzed using different protocols across laboratories. Most recently, microarrays by Affymetrix and Illumina have become widely used. While both platforms rely on DNA oligonucleotides as probes, they are fundamentally different in hybridization technology and data preprocessing protocols. Affymetrix arrays use in situ synthesis of 25-mer oligonucleotides while Illumina arrays are based on microbeads which self-assemble onto the array. Each Affymetrix probe is therefore hybridized to a predefined location [1] while the location of each probe on the Illumina array has to be determined using a molecular address [2]. Aside from physical differences, the two platforms also differ in the way in which probes are designed. In general, while Affymetrix uses multiple 25-mer probes for each gene, Illumina uses, on average, 30 copies of the same 50-mer probe (bead-type) for each gene. Finally, while Affymetrix arrays are processed individually, Illumina arrays contain multiple arrays on a single chip, thus allowing for parallel processing. These differences have resulted in challenges in comparing data sets across platforms and across laboratories using different platforms.

A number of prior studies have been done in an attempt to evaluate the comparability of these and other microarray platforms [3-6]. These studies have mainly focused on comparing two very different samples such as different tissues [3,5], tumors [4], and treatment effects on tumors [6]. In this paper, we perform a cross-platform comparison on a single tissue type over time, namely, fetal lung tissue as a function of gestational age. The sample group used in this study is more closely related to experimental settings in which the differences among groups are not large, hence we do not expect large differences in expression among samples. However, this allows us to evaluate the robustness of the effects of different factors on cross-platform comparability in the presence of subtle differences among samples. To do so, we perform both statistical and functional analyses to evaluate for statistical comparisons, as well as, biologically relevant effects. We found that the correlation between the Affymetrix and Illumina platforms at the individual gene level is related to expression level, probe overlap, and p-value ranking within each platform and that the comparability is further improved when considered on a gene-set level using GO categories and KEGG pathways.

## Results

### Performing probe matching reduces the discrepancy between Affymetrix and Illumina platforms

In the following results and discussion, we will refer to unique probe sequences as "probes". In the Illumina platform, there are multiple copies of each bead-type (corresponding to a probe sequence) that has been summarized into a single probe expression by Illumina's BeadStudio software. For simplicity, we will refer to each bead-type as "probe". If no probe matching was taken into account and all probes (i.e. probe sequences) in both the Illumina chips and Affymetrix chips were used, then there was a large (nearly 15-fold) discrepancy between the number of significant genes, i.e. differentially expressed genes, in Illumina (n = 679) compared to Affymetrix (n = 10074), much larger than the ratio of the number of Affymetrix probe sets to number of Illumina probes (2.3-fold). In order to isolate the platform-dependent effects that are independent of the different sets and number of genes for each chip, we created a one-to-one mapping between Affymetrix and Illumina chips based on sequence mapping to RefSeq transcripts (see Methods for details).

**Figure 1:**
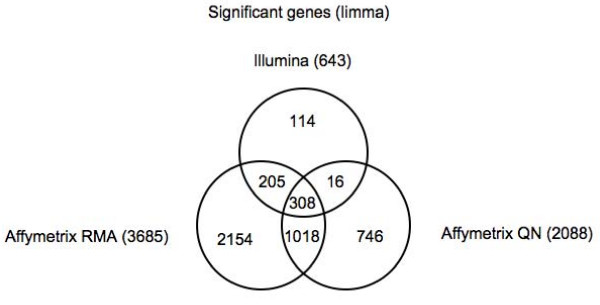
**Number of differentially expressed genes in Illumina and Affymetrix**. Affymetrix RMA was preprocessed using the Robust Multichip Average (RMA) algorithm. Affymetrix QN and Illumina were normalized using quantile normalization.

With probe matching, the discrepancy in the number of significant genes decreased (Figure 1). Affymetrix chips are commonly preprocessed with the Robust Multichip Average (RMA) algorithm [7]. This includes a summarization step that is unique to Affymetrix chips in which the multiple probe intensities within each probe set are combined to obtain an expression value for that probe set. In order to see what the effects of having multiple probes and a different normalization scheme would be, i.e. RMA applied to the Affymetrix chips, we compared the Illumina chips using quantile normalization to Affymetrix normalized using both RMA (Affymetrix RMA) and quantile normalization (without summarization) of the best-matched probes in the Affymetrix probe sets (Affymetrix QN). The use of the Affymetrix QN preprocessing scheme also gives us an idea regarding the effects of the different normalization schemes and what the "best" case scenario could be when comparing Illumina and Affymetrix chips. After probe mapping, the discrepancy between the number of Affymetrix (RMA n = 3685; QN n = 2088) and Illumina (n = 643) significant genes is reduced to 5.7-fold and 3.2-fold, respectively.

### To perform the cross-platform comparisons, we used differentially expressed genes and gene expression correlation as measures of comparability

In order to perform cross-platform comparisons, we used two different measures of similarity. The first measure of comparability is gene-wise correlation, a commonly used means of comparing different platforms. High correlation implies good comparability of two platforms for that gene regardless of whether or not it is differentially expressed. The second measure of comparability is the similarity or differences in the statistically significant differentially expressed genes. There are 4 groups of genes: 1) genes that demonstrate statistically significant differential expression with lung development on both platforms (common significant genes, G_ai_), 2) genes that are significantly differentially expressed on Illumina but not Affymetrix (G_i_), 3) genes that are significantly differentially expressed on Affymetrix but not on Illumina (G_a_), and 4) genes that are not significantly differentially expressed on either platform (G_ns_). We will refer to these 4 categories as significance groups. The difference between the two platforms lies mainly in the two groups in which genes are significant in one platform but not in the other. Two perfectly compatible platforms will only have genes in G_ai _and G_ns _and no gene in G_a _or G_i_. When two platforms are not perfectly comparable, knowing what features predicts which significance category a gene belongs to allow us to interpret results of one platform without having data from the other platform.

### Performing single probe matches between Affymetrix and Illumina using quantile normalizations does not significantly change the comparability of the two platforms compared to using RMA normalization for Affymetrix

**Figure 2:**
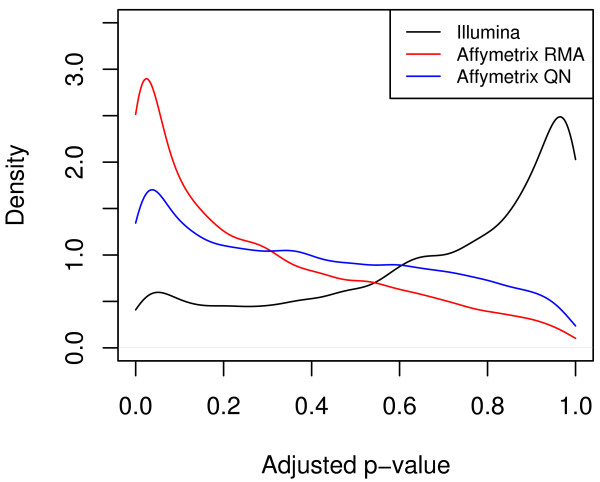
**Distribution of adjusted p-values for Illumina, Affymetrix RMA, and Affymetrix QN**. P-values have been adjusted for multiple testing via the Benjamini and Hochberg method. Prior to adjusting for multiple testing, the distributions were similar in appearance with a lower density of Illumina p-values near 0 compared to Affymetrix. The relatively lower density of Illumina adjusted p-values near 0 explains why there are many more differentially expressed genes in Affymetrix compared to Illumina.

Illumina and Affymetrix RMA identified 513 differentially expressed genes in common, whereas 324 common significant genes were identified between Illumina and Affymetrix QN. Part of the reason for the larger number of significant Affymetrix genes can be seen from the adjusted p-value distribution (i.e. p-value after correcting for multiple testing) (Figure 2). While most Illumina genes have high adjusted p-values, most Affymetrix genes have low p-values. The normalization method in Affymetrix does not affect the overall distribution pattern of the p-values, however. The mean Spearman correlation between Illumina and Affymetrix RMA is 0.22 and that between Illumina and Affymetrix QN is 0.15. The low correlation is somewhat lower than some prior studies in which the correlation between Affymetrix and Illumina platforms was closer to 0.5 [8,9]. The reason for such a low correlation is the large proportion of nonsignificant genes in both platforms, which is a property of the biological system, studied, that is, the changes in a single organ over time. As will be shown below, genes that are significant in both platforms have a much higher correlation. The correlation between the two Affymetrix normalizations is higher at 0.49. From these initial results, we see that the two different normalizations for Affymetrix do not lead to large differences in correlation between Affymetrix and Illumina platforms. Restricting the Affymetrix probes to only the best matched probes rather than using RMA normalization does not lead to increased correlation or increased overlap in significant in genes with Illumina. Thus the main differences between Affymetrix and Illumina cannot be accounted for by using the same normalization scheme for both. We will therefore restrict the remainder of our analysis to the comparison of Affymetrix RMA and Illumina, which utilize commonly used normalization methods for both platforms.

### Genes that are identified as differentially expressed in both platforms have the highest correlations, while genes that are identified as differentially expressed in only one platform have intermediate correlations and genes that are not identified as differentially expressed in any platform have the lowest correlations

**Figure 3:**
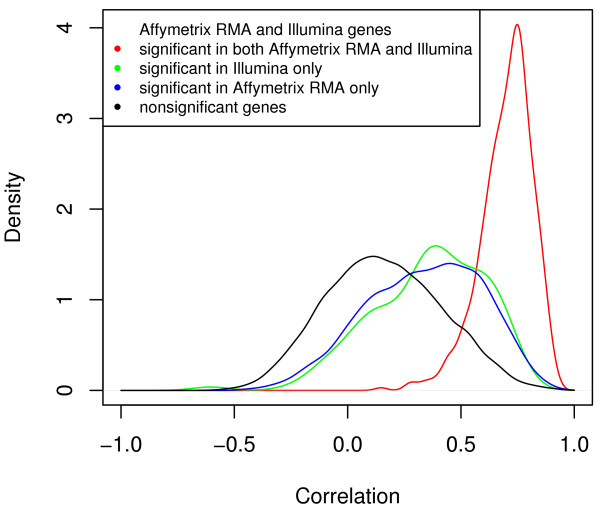
**Distribution of gene-wise correlations between Affymetrix RMA and Illumina**. Genes were divided into four groups: genes that were significant (differentially expressed) in both Affymetrix and Illumina (G_ai_), genes that were significant in Illumina only (G_i_), genes that were significant in Affymetrix only (G_a_), and genes that were not significant in either platform (G_ns_). The mean correlations for the four groups were 0.700, 0.358, 0.337, and 0.160, respectively. The variances of the distributions for the four groups were 0.013, 0.060, 0.065, and 0.065, respectively. Using the t-test, G_ai _is significantly different from G_i _and G_a _(p < 2.2 × 10^-16 ^for both). Similarly, G_ns _is significantly different from G_i _and G_a _(p = 9.463 × 10^-16 ^and < 2.2 × 10^-16^). The correlations were highest in genes that were significant in both platforms and lowest in genes that were insignificant in both platforms.

First we examined the relationship between the two measures we are using for comparison: correlation and significance class (Figure 3). Genes that are common to both Affymetrix and Illumina, G_ai_, have very high correlation of 0.70 ± 0.12 and a narrow distribution. Genes that are significant in Illumina only, G_i_, have correlations of 0.36 ± 0.24 and those that are significant on Affymetrix only, G_a_, have correlations of 0.34 ± 0.25. Genes that are not significant in either platform, G_ns_, have a low correlation of 0.16 ± 0.26. Note that the distribution is much broader for G_i_, G_a_, and G_ns_. Using the t-test, the differences in correlation between G_ai _and both G_i _and G_a _are significant (p < 2.2 × 10^-16^). Similarly, genes that are not significant in either platform, G_ns_, are also significantly different from G_i _and G_a _(p = 9.5 × 10^-16 ^and < 2.2 × 10^-16^, respectively). Thus while the correlations of genes that are significant in only one platform is much smaller than correlations of genes that are significant in both platforms, significance in at least one platform distinguishes a gene from genes that are not significant in any platform. In addition, this relationship demonstrates that the two measures of comparability that we are using, namely, significance class and correlation, are consistent but not the same.

Our goal is to determine factors that will let us predict which genes are consistently identified as differentially expressed between the platforms, either by predicting the significance category to which individual genes belong or by predicting its level of correlation with the other platform. We will examine both of these factors with respect to expression level, probe overlap/distance, and the significance rank of genes.

### Expression level, probe distance, and p-value rankings are associated with highly correlated genes and genes in G_ai_

We demonstrate in the sections below the associations between expression level, probe distance, and p-value rankings with correlation and significance categories (Tables 1 and 2). Although there were statistically significant associations found for all three features, the distribution of each over correlation and significance categories was very broad, which is likely reflective of the biological system studied, that is, subtle changes that occur over gestational age.

**Table 1:** Summary of single gene analysis for genes in different significance groups and with high/low correlations

	**High correlation (≥ 0.5)**	**Low correlation (< 0.5)**	**G_ai_**	**G_i_**	**G_a_**	**G_ns_**
Mean expression	7.703 ± 1.890	6.467 ± 2.280	7.358 ± 1.818	5.579 ± 1.611	7.609 ± 2.059	6.412 ± 2.269
Mean probe distance	64.8 ± 375	208.1 ± 951	109 ± 464	251 ± 785	220 ± 1475	175 ± 643
Median probe distance	-25	-21	-25	-20	-22	-22
Mean Illumina p-value rank	3956 ± 3817	8466 ± 4140	306 ± 186	385 ± 170	6277 ± 4424	8469 ± 4069
Mean Affymetrix p-value rank	4664 ± 4244	8315 ± 4200	915 ± 915	7763 ± 3548	1993 ± 1009	9537 ± 3360

**Table 2:** Summary of mean correlation in single gene analysis

	**Mean correlation**	P-value*
High Illumina expression (≥ 6)	0.296 ± 0.264	p < 2.2 × 10^-6^
Low Illumina expression (< 6)	0.099 ± 0.252	
High Affymetrix expression (≥ 6)	0.279 ± 0.266	p < 2.2 × 10^-6^
Low Affymetrix expression (< 6)	0.136 ± 0.269	
Perfect match	0.251 ± 0.280	p_perfect match, partial overlap _= 6.097 × 10^-10^
Partial overlap	0.215 ± 0.278	p_perfect match, no overlap _< 2.2 × 10^-16^
No overlap	0.169 ± 0.265	p_partial overlap, no overlap _= 3.202 097 × 10^-14^

* p-values were obtained using the t-test

### Expression level

Genes with low expression are generally thought to lack statistical significance as their signals cannot be readily distinguished from noise. As a result, many expression analyses are performed with low expression genes filtered out. One may therefore anticipate that high expression genes are more comparable between platforms. We examined the effect of gene expression level on the comparability of the two platforms via gene-gene correlation and significance category.

**Figure 4:**
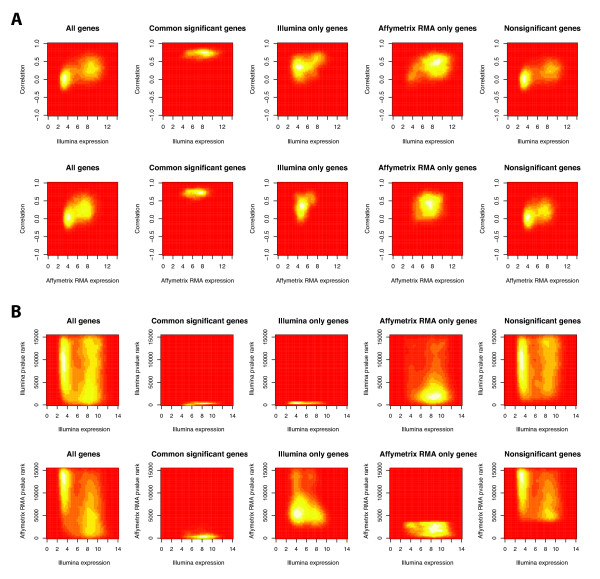
**Associations between expression level and gene-wise correlation and significance group**. A) Two-dimensional density plots of expression value versus correlation in different significance groups. B) Two-dimensional density plots of Illumina expression value versus p-value ranks in different significance groups. The density plot for Affymetrix expression value versus p-value ranks is shown in Additional File 1. There are proportionately more genes in G_ns _in the low-expression group compared to the high expression group. The converse is true for G_ai_. The density plots were obtained via the MASS package in R [10]. The bimodal distribution is most noticeable in G_ns_.

The distribution over mean Illumina and Affymetrix expression versus gene-gene correlation demonstrates that genes with high correlations tend to have higher levels of expression (Figure 4, Additional File 1) [10]. High and low correlation genes (corr ≥ 0.5 and <0.5) have significantly different mean expressions of 7.703 ± 1.890 and 6.467 ± 2.280, respectively (Wilcoxon rank sum, p < 2.2 × 10^-6^). However, the distributions are very broad with the distribution for low correlation genes being bimodal. Conversely, genes with high expressions (≥ 6) in either platform have higher correlations. The mean correlations for highly expressed and lowly expressed Illumina genes are 0.296 ± 0.264 and 0.099 ± 0.252 (t-test, p-value < 2.2 × 10^-6^). Similarly, the mean correlations for highly and lowly expressed Affymetrix genes are 0.279 ± 0.266 and 0.136 ± 0.269 (t-test, p-value < 2.2 × 10^-6^). Of note, although high expression does imply higher correlation, the level of correlation for highly expressed genes is not as high as that of the genes in G_ai_.

Similarly, we examined the distribution over expression levels of Affymetrix and Illumina in different significance categories (Figure 4, Additional File 1). Although G_ai _and G_ns _have distinct distributions with respect to expression levels, the distribution for G_ns _is bimodal and both distributions are very broad. The distribution of the G_i _and G_a _genes are similarly broad with G_i _more similar to G_ns _and G_a _more similar to G_ai_. Furthermore, although genes in G_ai _are associated with higher expression, so are genes in G_a_. Thus, while t-tests revealed the mean expressions of G_a _and G_i _to be statistically different from both G_ai _and G_ns_, the broad distributions and the similarly high expression of G_a _genes prevents using expression levels as a practical tool in predicting which significance group a gene belongs to.

### Probe distance

We now turn to probe matching or overlap as a means of predicting a gene's significance group and cross-platform correlation. Intuitively, we expect that probes that correspond to the same segment of a gene will have better cross-platform comparability. We had defined perfectly matched probes to be the Affymetrix probeset that contains a probe whose sequence matches that of the corresponding Illumina probe maximally (see Methods for details). Because Illumina probes are 50-mers and Affymetrix probes are 25-mers, the maximum probe overlap is 25. Using these criteria, 45% (6855 out of 15348) are considered perfectly matched.

**Figure 5:**
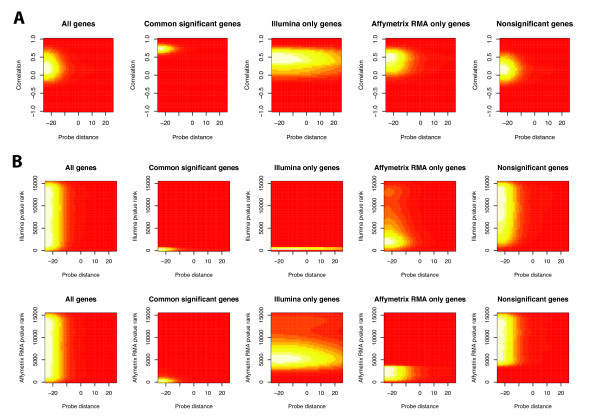
**Associations between probe distance between the best-matched Affymetrix and Illumina probes and their significance group and correlation**. A) Two-dimensional density plots of probe distance versus correlation in different significance groups. B) Two-dimensional density plots of probe distance versus p-value ranks in different significance groups. Distance less than 0 indicates overlap between probes. Probes corresponding to genes in G_ai _and G_ns _have the greatest overlaps.

The overall trend of correlation and probe distance in each significance category is shown in Figure 5 and Additional File 2. Highly correlated genes (corr ≥ 0.5) have higher overlap (lower distance) with the mean probe distance of 64.8 ± 375, while genes with low correlations (corr < 0.5) have lower overlap (higher distance) with a mean probe distance of 208.1 ± 951 (t-test, p-value < 2.2 × 10^-6^). Conversely, when we examined the correlation distribution of genes based on their overlap, we found that genes with perfectly matched probes have the highest correlations (corr = 0.251 ± 0.280), while genes with partially overlapped probed have lower correlations (corr = 0.215 ± 0.278), and genes with no overlap have the lowest correlations (corr = 0.169 ± 0.265). Although the differences in mean correlation of each group of genes based on overlap/distance are statistically significant, the large variances of the distributions and the small differences in means renders the degree of probe overlap ineffective as a predictor of cross-platform correlation.

The distribution of each significance category over probe overlap/distance demonstrates that genes that are common in significance to both platforms, G_ai _and G_ns_, have higher overlaps, than those that differ between platforms, G_i _and G_a _(Figure 5, Additional File 2). The mean interplatform probe distance for G_ai_, G_i_, G_a_, and G_ns _are 109, 251, 220, and 175, respectively. The distributions are narrowest for G_ai_. Thus higher overlap/lower distance seems to be associated with the G_ai _significance group. When we perform a χ^2 ^test between overlap and significance group, we found that there was an association between the two factors (p = 0.006). However, restricting the set of probes to perfectly matched probes does not change the fraction of genes that are in each significance group sufficiently to be a useful tool for discriminating between significance groups.

### P-value ranking

The p-value profile of Illumina and Affymetrix (Figure 2) suggests that a reason for the low correlation may be the difference in threshold for significance because of a specific p-value cutoff. To circumvent the p-value cutoff, we will examine the p-value rankings in each platform as a predictor of highly correlated genes and of significance class.

**Figure 6:**
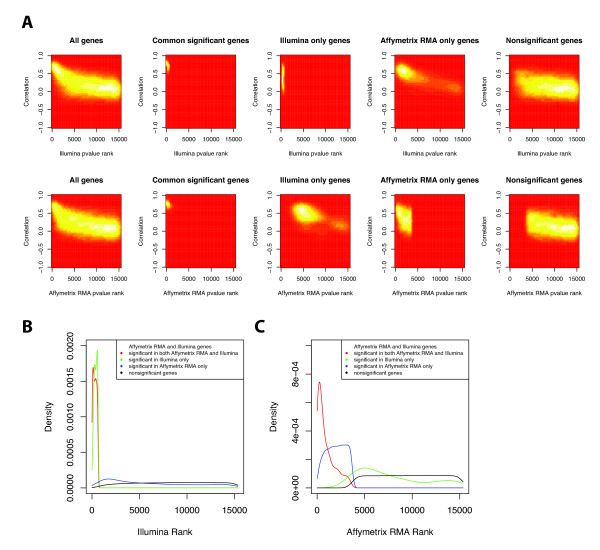
**Associations between p-value rankings and significance groups and correlation**. A) Two-dimensional density plot of correlation versus p-value ranking (Illumina and Affymetrix) for different significance groups. B, C) Genes in G_ai _and G_ns _have the lowest and highest p-value rankings for both Affymetrix and Illumina while those in G_i _and G_a _have intermediate rankings. The mean Illumina ranks for G_ai_, G_i_, G_a_, and G_ns _are 306, 385, 6277, and 8469, respectively. The mean Affymetrix ranks for G_ai_, G_a_, G_i_, and G_ns _are 915, 1993, 7763, and 9537, respectively. Using the Wilcoxon rank sum test, the Illumina p-value ranking for G_ai _is significantly different from G_i _and G_a _(p = 1.517 × 10^-5 ^< 2.2 × 10^-6^). Similarly, the Illumina p-value ranking for G_ns _is significantly different from G_i _and G_a _(p < 2.2 × 10^-16 ^for both). The Affymetrix p-value ranking for G_ai _is significantly different from G_i _and G_a _(p < 2.2 × 10^-16 ^for both). Similarly, the Affymetrix p-value ranking for G_ns _is significantly different from G_i _and G_a _(p = 2.338 × 10^-9 ^and < 2.2 × 10^-16^).

Genes with high correlation are associated with lower ranks in both Illumina and Affymetrix (Figure 6, Additional File 3). The mean Illumina ranks of genes with high and low correlations are 3956 and 8466, and the mean Affymetrix ranks of genes with high and low correlations are 4664 and 8315. Both differences are statistically significant with Wilcoxon rank sum p-values of < 2.2 × 10^-6^. Of note, the mean Affymetrix ranks for genes with high and low correlations are very similar to mean Illumina ranks for those genes.

When we examine the significance of the genes with respect to their p-value ranking in one platform, we find that those genes, which are significant only in the other platform, tend to have lower ranks. The mean Illumina ranks for G_ai_, G_i_, G_a_, and G_ns _are 306, 385, 6277, and 8469, respectively (Figure 6). The mean Affymetrix ranks for G_ai_, G_a_, G_i_, and G_ns _are 915, 1993, 7763, and 9537, respectively. The difference in mean Illumina ranks between G_ai _and G_i _is much less than the difference in mean Affymetrix ranks between G_ai _and G_a_. Using the Wilcoxon rank sum test, we found that both G_i _and G_a _are statistically different from G_ai _and G_ns _for both Illumina and Affymetrix rankings. In particular, when examining Illumina ranks, G_i _is statistically different from G_ai _with a p-value of 1.517 × 10^-5^, and G_a _statistically different from G_ns _with a p-value of < 2.2 × 10^-6^. Similarly, when examining Affymetrix ranks, G_a _is statistically different from G_ai _with a p-value of < 2.2 × 10^-6^, and G_i _statistically different from G_ns _with a p-value of 2.338 × 10^-9^. These distributions demonstrate that the common significant genes, G_ai_, have the lowest rankings (most significant). More interestingly, we also see that the genes that are significant in only one platform have the next higher ranking in that platform. It is also important to note that although genes that are significant only in the other platform are distributed throughout the rankings, they are concentrated at the lower rankings when compared to nonsignificant genes.

### Gene sets using GO categories and KEGG pathways are more consistent between platforms than the number of significant individual genes

Significant GO categories and KEGG pathways were determined using SAFE [11] correcting for multiple testing via the method of Westfall and Young [12]. However, correcting for multiple testing yielded no significant results. We therefore proceeded to examine the results based upon the empirical p-values alone. This is similar to analyzing the gene sets based on gene rankings.

The effects of consolidating genes into gene sets can be seen even before probe matching. While the number of significant genes in Affymetrix RMA and Illumina differ by nearly 15-fold when examining single genes, grouping the genes attenuates the discrepancy. There are approximately twice as many significant GO categories and KEGG pathways in Affymetrix RMA than in Illumina prior to probe matching.

**Figure 7:**
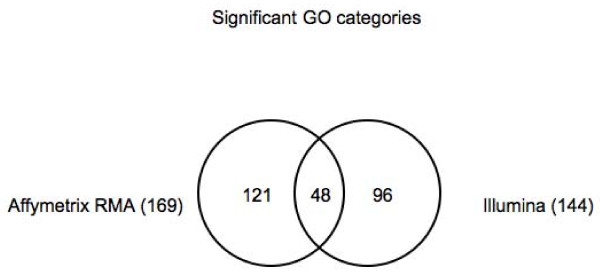
**Number of significant GO categories in Affymetrix and Illumina**.

**Figure 8:**
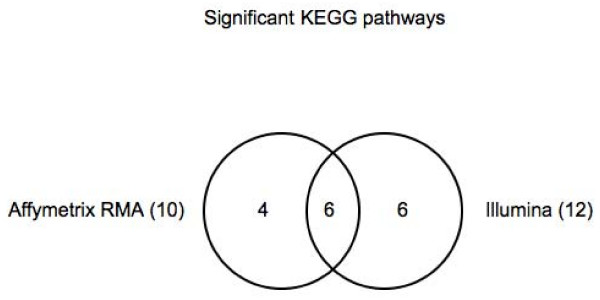
**Number of significant KEGG pathways in Affymetrix and Illumina**.

Probe matching reduces the discrepancy even further with the number of GO categories and KEGG pathways for the two platforms being within 20% of each other (Figures 7, 8). GO categories are not as effective as KEGG pathways, however, in capturing the same biology between the two platforms. While only about 30% of the significant GO categories are common to the two platforms, about 50% of the KEGG categories are. However, if we analyze the ancestral terms of the GO categories as derived from the GO slim subset obtained via cateGOrizer (, December 3, 2008) [13], counting only one path between a parent and child term, we found that the ten most frequent ancestral terms are the same between Affymetrix and Illumina (Additional File 4). There was only one significant GO category in Illumina that was not part of GO slim, GO:003326, and two terms in Affymetrix that were not part of GO slim, GO:0022884 and GO:0033261.

### The proportions of significant GO categories are similar in Illumina and Affymetrix but significantly different from the distribution of all GO categories

**Figure 9:**
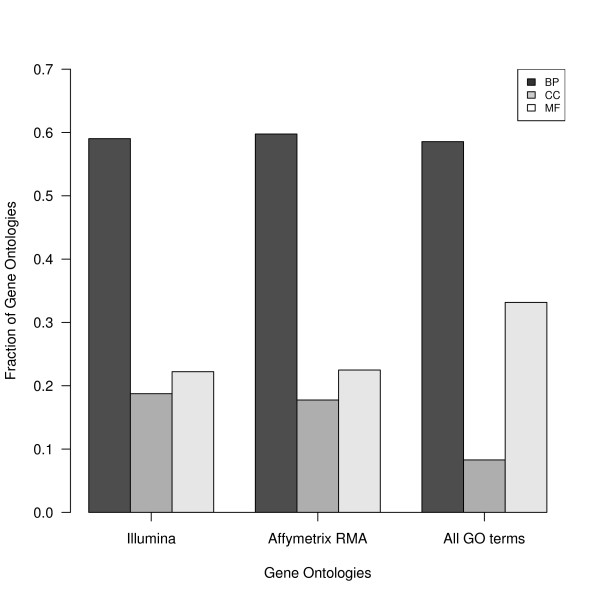
**Distribution of GO categories in Illumina and Affymetrix compared to all GO terms**. The proportion of significant GO categories under cellular components is more than twice as high in Affymetrix and Illumina than over all GO terms while the proportion of categories under molecular function is less than 2/3 compared to all GO terms. CC = cellular component, BP = biological processes, MF = molecular function.

While the fraction of biological processes (BP) in Illumina and Affymetrix is very similar to that over all GO terms, the proportion of significant GO terms in cellular components (CC) is more than twice as high in Illumina and Affymetrix than over all GO terms (0.19 and 0.18 vs. 0.08) while the fraction of molecular function (MF) is less than 2/3 of all GO terms (0.22 and 0.22 vs. 0.33) (Figure 9). Using Pearson's χ^2 ^test over all 3 categories, both Illumina and Affymetrix are significantly different from the set of all GO terms (p = 6.19 × 10^-6 ^and 6.6 × 10^-6^), whereas the difference between Affymetrix and Illumina is not significantly different from each other (p = 0.9743).

### Most locally significant genes in significant KEGG pathways or GO categories in Illumina are also significant in Affymetrix. These common significant genes are highly correlated

**Figure 10:**
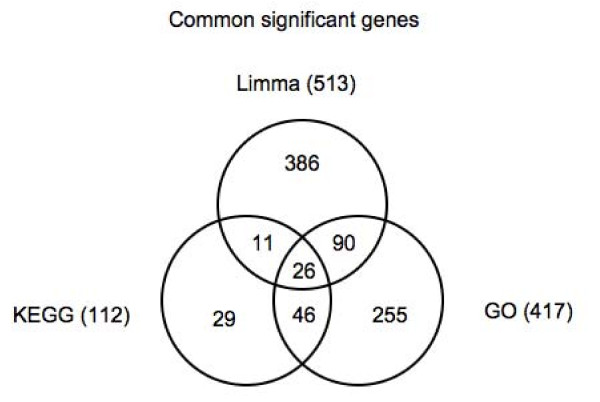
**Number of significant genes common to both Affymetrix and Illumina that were obtained via a categorical linear model (SAFE **[11]**package from Bioconductor), from significant KEGG pathways and from significant GO categories**.

There are 6 common significant KEGG categories for Illumina and Affymetrix and 48 common significant GO categories (Additional File 5). For each category, we examined the locally significant genes. 78% and 83% of the genes significant in Illumina are also significant in Affymetrix over all KEGG pathways and GO categories, respectively. Because there are slightly more significant genes in Affymetrix (213 and 1562) than Illumina (190 and 1289) in the significant KEGG pathways and GO categories, the percentage of genes significant in Affymetrix that are also significant in Illumina is smaller (69% and 68%). There are 148 and 1066 such common significant genes identified in the KEGG pathways and GO categories, respectively. Since some of these genes are in multiple KEGG pathways, the number of unique common significant genes is 112 (KEGG) and 417 (GO). These common significant genes as determined from significant KEGG pathways are highly correlated between Illumina and Affymetrix with a correlation of 0.59 (KEGG) and 0.55 (GO). Of note, these are not the same genes as those found using single gene analysis. In fact, only 37 of the 112 genes in the KEGG pathways and 116 of 417 genes in the GO categories also belong to the common significant genes from the single gene analysis (Figure 10). Similarly, there is an overlap of 72 genes between the common significant genes derived from the GO and KEGG pathways. Because of the large proportion of genes that are significant in only one analysis, that is, linear regression, GO categories, or KEGG pathways, common significant genes between Affymetrix and Illumina from each analysis are not predictive of the common significant genes in the other two analyses.

### qPCR results of G_ai _and G_ns _genes were consistent with Affymetrix and Illumina gene expression

To validate results from the microarrays, the same analysis was done using the piecewise constant model on ΔCt from qPCR results from Kho et al (Kho AT, Bhattacharya S, Tantisira KG, Carey VJ, Gaedigk R, Leeder JS, Kohane IS, Weiss ST, Mariani TJ: Transcriptomic Analysis Identifies Molecular Phases of Human Lung Development, unpublished). Genes that were significant in both Affymetrix and Illumina were also significant by qPCR (Additional File 6).

## Discussion

Given the emergence of multiple platforms for performing microarray analysis, many cross-platforms studies have been done in order to identify characteristics in the platforms that will allow the comparison of data, and possibly the ability to combine data from different platforms. Most such studies involve two very distinct contrasting groups. In this study, we performed a cross-platform analysis across samples that differ with respect to gestational age to evaluate the robustness of cross-platform comparisons under subtle changes in biological conditions. When compared to other studies that have interrogated both Affymetrix and Illumina platforms, including the MicroArray Quality Control (MAQC) project, we noted in our biologic model a disproportionate representation of significant Affymetrix probes, when compared to Illumina. Despite these differences, detection of ontologic pathways was similar in both platforms, suggesting that both platforms suitably detect the same underlying biologic processes.

The discrepancy between Affymetrix and Illumina chips prior to probe mapping is striking in our dataset with a nearly 15-fold difference in the number of differentially expressed genes over gestational age. Limiting the analysis to matched probes between the two platforms largely decreases this discrepancy. To confirm that this discrepancy is present even when done using a different preprocessing method, we applied cubic spline normalization to Illumina and probe logarithmic intensity error estimation (PLIER) to Affymetrix data (data not shown), as done in the MAQC project [5]. This approach did not significantly impact the relative preponderance of Affymetrix significant genes.

Our goal was then to perform the cross-platform comparison using common preprocessing schemes for each type of chip, namely, RMA for Affymetrix chips and quantile normalization for Illumina chips, so that our results would be applicable to the situation commonly encountered in practice when comparisons need to be made between laboratories. However, since these two preprocessing methods are considerably different, we first assessed the effects of performing the exact preprocessing methods on both types of chips.

If we were to take the Affymetrix genes and normalize them in the same way as RMA but without the summarization, we would have a normalization scheme that one often uses on Illumina chips in which RMA summarization is not applicable. In this way, the quantile normalized (but not summarized) Affymetrix data can serve as the most comparable way by which we can compare the Illumina data to the Affymetrix data. We found the correlation between Illumina and Affymetrix chips to be low, regardless of the normalization used on Affymetrix. The correlation is much lower than that between the two Affymetrix normalizations. The correlation between Affymetrix quantile normalization and RMA demonstrates how the difference in normalization schemes is sufficient to reduce the correlation to less than 0.5. However, the difference between Affymetrix and Illumina is more than just the normalization scheme. Even with sequence-based probe matching and the same normalization scheme, the correlation between Affymetrix and Illumina remains low.

One reason for the difference between Affymetrix and Illumina can be seen from the p-value distribution of the two platforms. While most genes in Illumina have very high p-values, most genes in Affymetrix have very low p-values. This also explains why most significant Illumina genes are a subset of the significant Affymetrix genes but not vice versa. This is consistent with Illumina being more specific and Affymetrix being more sensitive, similar to findings of Chen et al [14]. The difference in platforms accentuates the need for cross-platform comparisons. Our goal was to evaluate which features of the Affymetrix and Illumina platforms would give us the most comparable and consistent data between the two platforms.

In order to compare the two platforms for compatibility, we used two different measures for quantifying the "distance" between platforms, namely, the significance categories and gene-wise correlation. The significance categories divide genes into those which are significantly dependent on gestational age in both platforms, not significant in either platform, or significant in one platform but not the other. These categories are different from but complementary to gene-wise correlations as one is categorical while the other is continuous. If the correlation distribution of each significance group were narrow and distinct, the two measures of platform comparability would approach one another.

One reason for the low overall correlation between the platforms is the presence of a large number of genes that are not differentially expressed during lung development. Restricting the set of genes to G_ai _versus G_i _or G_a _increases the correlation by variable amounts. As one would expect, genes in G_ai _have the highest correlations while those in G_ns _have the lowest correlations. The distribution of correlation is very broad, however. Therefore, we have used both measures in our analyses.

A common practice in microarray analysis is the filtering out of genes with low expression values [15-17]. The effects of filtering were evaluated by retaining genes with median absolute deviations in the top 20% (data not shown). Again, a larger number of significant Affymetrix genes remained despite the filtering. Our final analysis, therefore, was based on unfiltered data. This also allowed us to evaluate the contribution of the low expression genes. Barnes et al. have previously shown that correlation between genes is associated with higher expression levels [3]. While we found that the mean expression for genes in G_a _and G_i _are statistically different from those in G_ai _and G_ns_, the expression distribution is so broad and sometimes bimodal that one cannot use expression to predict significance group. The same is true for using expression to predict correlation. On the other hand, genes with low correlation or those in G_ns _have a bimodal distribution over expression. This means that while filtering does preferentially eliminate genes that are nonsignificant or have low correlation between the platforms, eliminating low expression genes will also eliminate some highly correlated or significant genes.

Mecham et al. previously found that the degree of probe overlap between two platforms is associated with correlation [18]. We found, as one would expect, that probes for genes in G_ai _and G_ns _have higher overlaps (lower distance) than those in G_a _and G_i_. Similarly, genes that are highly correlated between the two platforms also have higher overlaps. However, similar to the scenario with expression levels, the overlap/distance distributions are very broad. Thus while we have statistical difference in terms of correlation or significance group, we cannot use the degree of probe overlap/distance to predict highly correlated genes or their significance group using our dataset. This may improve as the number of samples increases, thereby giving higher power to the analysis.

Lastly, we had examined correlation and significance group with respect to p-value ranking. Although the rank distributions are more distinct between groups compared to overlap or expression distribution, they are also broad and cannot be used reliably to predict either correlation or significance group. We thus face similar issues when attempting to use probe overlap/distance, expression level, or p-value ranking in determining which genes are comparable across platforms despite obvious trends. The broadness of the distributions may be specific to our dataset, however. First, it is a relatively small sample size, especially with respect to each age group. With increased sample size, many of the variances of the different distributions may decrease, rendering each factor more predictive of a gene's cross-platform comparability. In addition, unlike most other cross-platform studies, we are examining gene expression as a continuous variable – a more gradual change in lung tissue gene expression over gestational age, rather than as a dichotomous variable – comparing two vastly different tissues such as normal versus tumor tissues, for example. There are two implications from assessing change over time. First, there is biological noise, that is, potential errors in reporting gestational age. Second, significant genes over time may follow a subtle trend rather than having two very different expression levels between two groups. This may result in the smaller differences in mean overlap/distance, expression, or p-value ranking than if we had very different control and experimental groups.

Although examining the lowest ranking genes in the Affymetrix platform can reduce the discrepancy between the Affymetrix and Illumina platforms at the single gene level, the cutoff is difficult to determine ahead of time. To address this problem, we performed a gene set (GO and KEGG) enrichment analysis to determine if the biologically relevant gene sets are comparable between the two platforms. Maouche et al. utilized a post hoc test for the relative enrichment of a gene category within the list of significant genes to look for biological function and relevance [19]. We used the SAFE package from Bioconductor which accounts for the unknown correlation among genes [11]. We found that consolidating genes into gene sets removes the large discrepancy in the number of significant genes as was found when single gene analysis was done. The number of significant categories is similar across the Illumina and Affymetrix platforms. This is true for grouping genes into gene sets as defined by KEGG pathways and ancestral terms of the GO categories (Additional File 4). The similar number of significant KEGG pathways and GO categories in both Affymetrix and Illumina and high percent of overlap between them (about 50%) implies a higher degree of consistency between the platforms when biological function is considered rather than single genes. The relative contribution of each gene in a category can be seen from the empirical distribution function of the ranked local statistics (statistics of a single gene considered independently of its category) (Additional File 7). Because the overall contribution of all genes within a category is taken into consideration, variations in p-values of individual genes are less likely to affect the significance of a category, thereby rendering gene set analysis more robust and less susceptible to cross-platform variation than single-gene analysis.

Conversely, most locally significant genes in each common significant KEGG pathways or GO categories in Illumina are also significant in Affymetrix and vice versa. Furthermore, these common significant genes as determined by the common KEGG pathways and GO categories are highly correlated. This supports the notion that the KEGG pathways and GO categories bring out not only the relevant biological functions but also the genes associated with them.

One should note, however, that the common significant genes arrived at via single gene analysis, via KEGG pathways and via GO categories are largely nonoverlapping. This explains why the distribution of correlation of the non-common significant genes from the single gene analysis is a bimodal distribution. Given the high correlation of these genes between two very different platforms, one cannot dismiss one set or the other. This brings out the complexity of the biology. There are genes that are best characterized as single genes, in terms of GO categories, and/or in terms of KEGG pathways. It emphasizes our incomplete understanding of the gene networks that are clearly not completely characterized by known KEGG pathways or GO categories. While the grouping of genes into gene sets creates a more consistent interplatform picture, the analysis should in no way be limited to any particular set.

## Conclusion

Although there are differences in the Affymetrix and Illumina technology and the number of differentially expressed genes in each platform, there are many comparable features between the two platforms. Gene-wise comparisons demonstrate that there is a relationship between expression level, probe overlap/distance, and p-value ranking with a gene's significance class. Functional analysis using GO categories and KEGG pathways show that despite the differences at the single gene level, the two platforms are very comparable in terms of biologically relevant variables. Neither platform can be clearly considered superior to the other based on this analysis.

## Methods

RNA was isolated from normal fetal human lung tissue samples obtained from two NICHD-supported tissue services, the Center for Birth Defects Research at the University of Washington, and the University of Maryland Brain and Tissue Bank for Developmental Disorders. The post mortem interval was <2 hr for all samples from the University of Washington and <6 hours for the samples from the University of Maryland. The collection of tissues was approved by the University of Missouri-Kansas City Pediatric Institutional Review Board. Gene expression data from 32 RNA samples from different stages of human lung development (53 days to 153 days estimated gestational age) were obtained for both the Affymetrix U133 Plus 2.0 (Affymetrix, Santa Clara, CA) and the Illumina HumanRef-8 v2 Expression Bead Chips (Illumina, San Diego, CA). Gene expression data for the Affymetrix chip was obtained from Kho et al [20]. The Affymetrix Human Genome U133 Plus 2.0 Array has 54,675 probe sets and 604,258 probes while the Illumina chip has 23,811 bead-types (each corresponding to a probe sequence). For simplification, we will refer to the Illumina bead-types as probes. Summarized, but unnormalized, bead-type data was obtained from Illumina's BeadStudio software. Expression sets for Affymetrix and Illumina were obtained via the affy [21] and lumi [22] packages from Bioconductor.

Validation of gene expression was done via quantitative PCR (qPCR) on a subset of 7 genes at 27 time points from 53 to 154 days of gestational age. The genes were selected for their association with immunologic response or surfactants, as well as, their significance (or lack thereof) in both Affymetrix and Illumina platforms. qPCR was performed on a Stratagene M X3000P using Taqman chemistry previously described in Simon et al [23]. Inventoried (pre-developed) gene-specific assays for measuring gene expression were obtained from Applied Biosystems. Gene expression levels (ΔCt) were obtained from Kho et al. and were calculated relative to the measured Ct value of PPIA (peptidyl prolyl isomerase A or cyclophilin A) as an internal, endogenous control [20]. For each gene, we computed the p-value for the ΔCt over gestational age using a piecewise constant model (see below).

### Probe mapping

In order to perform the cross-platform comparison, we obtained a one-to-one map between the Illumina probes and the Affymetrix probe sets. We started with the probe map provided by Illumina from HumanRef-6 v2 to Affymetrix HGU133 Plus 2.0 with RefSeq IDs (, Illumina Human-6v2 to Affymetrix U133Plus2.0). RefSeq sequences were obtained by querying NCBI Entrez Nucleotide on 5/21/2008. To obtain the initial probe map the manufacturer used transcript sequences from RefSeq Release 20. For both platforms, only probes that perfectly matched a unique RefSeq transcript were retained. Affymetrix probe sets were considered valid if at least 80% of the probes within the set were valid. If a transcript contained more than one probe or probe set, the one closest to the 3' end was retained. We verified the probes and probe sets provided by the manufacturer with the most recent RefSeq transcripts. Using the same criteria as the manufacturer, we found 221 mismatched Illumina sequences. After eliminating these sequences, there were 3 mismatched Affymetrix probe sets. In addition, there were two probe/probe sets that mapped to different RefSeq IDs.

Using the above probe map, we proceeded to probe-level matching of Affymetrix to Illumina probes. This was done by defining the distance between two probes as the distance apart subtracted by the overlap. Hence a positive distance implies no overlap and any overlap gives a negative distance. The best matching Affymetrix probe in the probe set was defined as the one with the maximum overlap with the Illumina probe. If there was no overlap, then the best-matched probe was the one with the minimum distance apart from the Illumina probe. If two Affymetrix probes had the same overlap or distance apart from the corresponding Illumina probe, then the probe that was closest to the 3' end was selected. Using the above algorithm, 15,348 matching Affymetrix-Illumina probe sets, as well as, the best matching probe within the Affymetrix probe set were obtained.

### Data Preprocessing

Two different normalizations were used for Affymetrix chips and one for Illumina chips. Robust Multichip Average (RMA) preprocessing was done over the entire Affymetrix chip set, followed by selection of the matching probe set. This expression data set will be referred to as Affymetrix RMA. A second normalization, which was done for both the best matched Affymetrix probes and the Illumina probes, involved background correction, log transformation, retaining PM only Affymetrix probes, and quantile normalization. The Affymetrix expression set normalized and probe-matched in this manner will be referred to as Affymetrix QN.

### Data Analysis

Because of distinct temporal expression patterns in fetal lung development [24], we divided the gestational age of the samples into 5 groups to allow for higher order age effects. To allow for more complex dependence on age, a piecewise constant model was used. Age was divided into five quantiles, based upon estimated days post-conception: (52.9,76], (76,87.8], (87.8,102], (102,121], and (121,154]. The expression values were then fitted to a piecewise constant mean value within each age group and evaluated using empirical Bayes moderated F-statistics using Bioconductor's limma package for a categorical linear model (Additional File 8) [25]. The Benjamini and Hochberg method was used to account for the multiple testing [26]. Significant genes were defined as those with adjusted p-values of less than 0.05. Spearman's correlation was applied. Unique RefSeq IDs which map to different transcripts of the same genes were counted as different genes.

Gene set enrichment analysis was done using the SAFE package in Bioconductor for both KEGG pathways (October 7, 2007 build) and GO categories (April 7, 2008 build), using the permutation method [11]. Multiple testing was done via Westfall and Young method with a family-wise error rate of 0.1. In the SAFE package, two kinds of statistics were obtained, local statistics and global statistics, which we will describe briefly below. The detailed description can be found in Barry et al [11]. Local statistics assesses the association between the expression of a single gene against age using linear regression. Global statistics measures how the distribution of local statistics of genes within a category (KEGG pathway or GO category) differs from the local statistics outside of the category, and is determined via the Wilcoxon rank sum statistic.

## Abbreviations

BP: biological processes; CC: cellular components; GO: gene ontology; KEGG: Kyoto Encyclopedia of Genes and Genomes; MAQC: MicroArray Quality Control; MF: molecular function; PLIER: probe logarithmic intensity error estimation; PPIA: peptidyl prolyl isomerase A or cyclophilin A; QN: Quantile Normalization; qPCR: quantitative polymerase chain reaction; RMA: Robust Multichip Average; SAFE: Significance Analysis of Function and Expression.

## Authors' contributions

RD was involved in the concept and design, data analysis, and manuscript writing and editing. KT was involved in concept and design, data collection, manuscript writing and editing. VC was involved in the data analysis, statistical support, and manuscript writing and editing. SB was involved in concept and design, data analysis, and data collection. SM was involved in data collection. ATK was involved in data collection. BJK was involved in data collection. RG was involved in data collection. RL was involved in data collection. TJM was involved in concept and design, and manuscript writing and editing. JSL was involved in data collection and manuscript writing and editing. STW was involved in concept and design, data collection, and manuscript writing and editing. All authors read and approved the final manuscript.

## Supplementary Material

Additional file 1 **Relationship between gene-wise correlation, significance group, and expression level**. A) Density plot over mean Affymetrix and Illumina expression level for high- and low-correlation genes. While highly correlated genes have higher expression overall, genes with low correlation have a bimodal distribution with respect to expression level. The mean expression levels for high- and low-correlation genes are 7.703 and 6.467, respectively. The difference in mean expression between high- and low-correlation genes is significant (p < 2.2 × 10^-16^, Wilcoxon sum rank test). B) Density of correlation in high- and low-expression genes. Genes that are highly expressed have higher correlation. The mean correlation for genes with Illumina expression ≥ 6 and < 6 are 0.296 and 0.099, respectively (p < 2.2 × 10^-16^, t-test). The variances of the high and low Illumina expression genes are 0.070 and 0.063. The mean correlation for genes with Affymetrix expression ≥ 6 and < 6 are 0.279 and 0.136, respectively (p < 2.2 × 10^-16^, t-test). The variances of the high and low Affymetrix expression genes are 0.071 and 0.072. C) Two-dimensional density plots of Affymetrix expression value versus p-value ranks in different significance groups. D) Distribution of mean expression level for each significance group. G_ai _and G_ns _have distinct but broad and overlapping distributions. The mean expression levels for G_ai_, G_i_, G_a_, and G_ns _are 7.358, 5.579, 7.609, and 6.412, respectively. The variances for G_ai_, G_i_, G_a_, and G_ns _are 3.30, 2.59, 4.24, and 5.15, respectively. Using the t-test, G_ai _is significantly different from G_i _and G_a _(p < 2.2 × 10^-16 ^and = 0.005). Similarly, G_ns _is significantly different from G_i _and G_a _(p = 3.836 × 10^-8 ^and < 2.2 × 10^-16^). E) Distribution of high- and low-expression genes over the significance groups. Click here for file

Additional file 2 **Relationship between gene-wise correlation and probe distance between the best-matched Affymetrix and Illumina probes**. A) Distribution of probe distance for high- and low-correlation genes. Genes that are highly correlated have greater probe overlap. The median probe distances for high- and low-correlation genes are -25 and -21, respectively (p < 2.2 × 10^-16^, t-test). B) Distribution over correlation for probes in which the best-matched Affymetrix and Illumina probes are perfectly matched, partially overlapped, and non-overlapping. Correlation increases as the degree of probe overlap increases. The mean correlation for the perfectly matched, partially overlapped, and non-overlapping probes are 0.251, 0.215, and 0.169, respectively. The variances are 0.078, 0.077, and 0.070, respectively. Using the t-test, p perfect match, partial overlap = 6.097 × 10^-10^, p perfect match, no overlap < 2.2 × 10^-16^, and p partial overlap, no overlap = 3.202 097 × 10^-14^. C) Distribution of probe distance for each significance group. The mean probe distance for G_ai_, G_i_, G_a_, and G_ns _are 109.1, 250.7, 219.5, and 175.4, respectively. The variances are 2.15 × 10^5^, 6.16 × 10^5^, 2.18 × 10^5^, and 4.14 × 10^5^, respectively. D) Distribution of best-matched probes that are perfectly matched, partially overlapped, and non-overlapping between Affymetrix and Illumina over significance group. Although the distributions over the different probe distances appear similar, the χ^2 ^test between probe distance and significance group showed an association between the two factors with p = 0.006. Click here for file

Additional file 3 **Distribution over p-value rankings for high- and low-correlation genes**. Highly correlated genes have lower p-value rankings with similar distributions in both Affymetrix and Illumina platforms. The mean Illumina p-value ranking for high- and low-correlation genes are 3956 and 8466 (p < 2.2 × 10^-16^, Wilcoxon sum rank test). The mean Affymetrix p-value ranking for high- and low-correlation genes are 4664 and 8315 (p < 2.2 × 10^-16^, Wilcoxon sum rank test). Click here for file

Additional file 4 **Top 10 GO_slim ancestor terms**. Comparison of GO_slim ancestor terms for Affymetrix and Illumina. Click here for file

Additional file 5 **Significant KEGG pathways**. Comparison of significant KEGG pathways between Affymetrix and Illumina. Click here for file

Additional file 6 **P-values of gene expressions in Affymetrix RMA, Illumina and p-values of ΔCt in quantitative PCR results**. CCL20, CXCL3, and CXCL5 were not significant in both Affymetrix and Illumina. CD36, SFTPB, SFTPC, and TUBB2B were significant in both Affymetrix and Illumina. Genes that were significant in both platforms were also significant in qPCR. Click here for file

Additional file 7 **Local statistics of genes in a significant KEGG pathway**. Empirical distribution function of the ranked local statistics of genes in a significant KEGG pathway (KEGG:04110) against that of all genes (A = Affymetrix, B = Illumina). Click here for file

Additional file 8 **Piecewise constant model for gene expression**. Affymetrix gene expression fitted to a piecewise constant model for two genes. Click here for file
