# Supplementary material for: Platform dependence of inference on gene-wise and gene-set involvement in human lung development
Source: BMC Bioinformatics. 2009 Jun 19;10:189. doi: 10.1186/1471-2105-10-189 (PMC2711081; doi:10.1186/1471-2105-10-189)
Supplement: Additional file 1 — Relationship between gene-wise correlation, significance group, and expression level. A) Density plot over mean Affymetrix and Illumina expression level for high- and low-correlation genes. While highly correlated genes have higher expression overall, genes with low correlation have a bimodal distribution with respect to expression level. The mean expression levels for high- and low-correlation genes are 7.703 and 6.467, respectively. The difference in mean expression between high- and low-correlation genes is significant (p < 2.2 × 10^-16^, Wilcoxon sum rank test). B) Density of correlation in high- and low-expression genes. Genes that are highly expressed have higher correlation. The mean correlation for genes with Illumina expression ≥ 6 and < 6 are 0.296 and 0.099, respectively (p < 2.2 × 10^-16^, t-test). The variances of the high and low Illumina expression genes are 0.070 and 0.063. The mean correlation for genes with Affymetrix expression ≥ 6 and < 6 are 0.279 and 0.136, respectively (p < 2.2 × 10^-16^, t-test). The variances of the high and low Affymetrix expression genes are 0.071 and 0.072. C) Two-dimensional density plots of Affymetrix expression value versus p-value ranks in different significance groups. D) Distribution of mean expression level for each significance group. Gai and Gns have distinct but broad and overlapping distributions. The mean expression levels for Gai, Gi, Ga, and Gns are 7.358, 5.579, 7.609, and 6.412, respectively. The variances for Gai, Gi, Ga, and Gns are 3.30, 2.59, 4.24, and 5.15, respectively. Using the t-test, Gai is significantly different from Gi and Ga (p < 2.2 × 10^-16^ and = 0.005). Similarly, Gns is significantly different from Gi and Ga (p = 3.836 × 10^-8^ and < 2.2 × 10^-16^). E) Distribution of high- and low-expression genes over the significance groups. [file 1471-2105-10-189-S1.pdf]

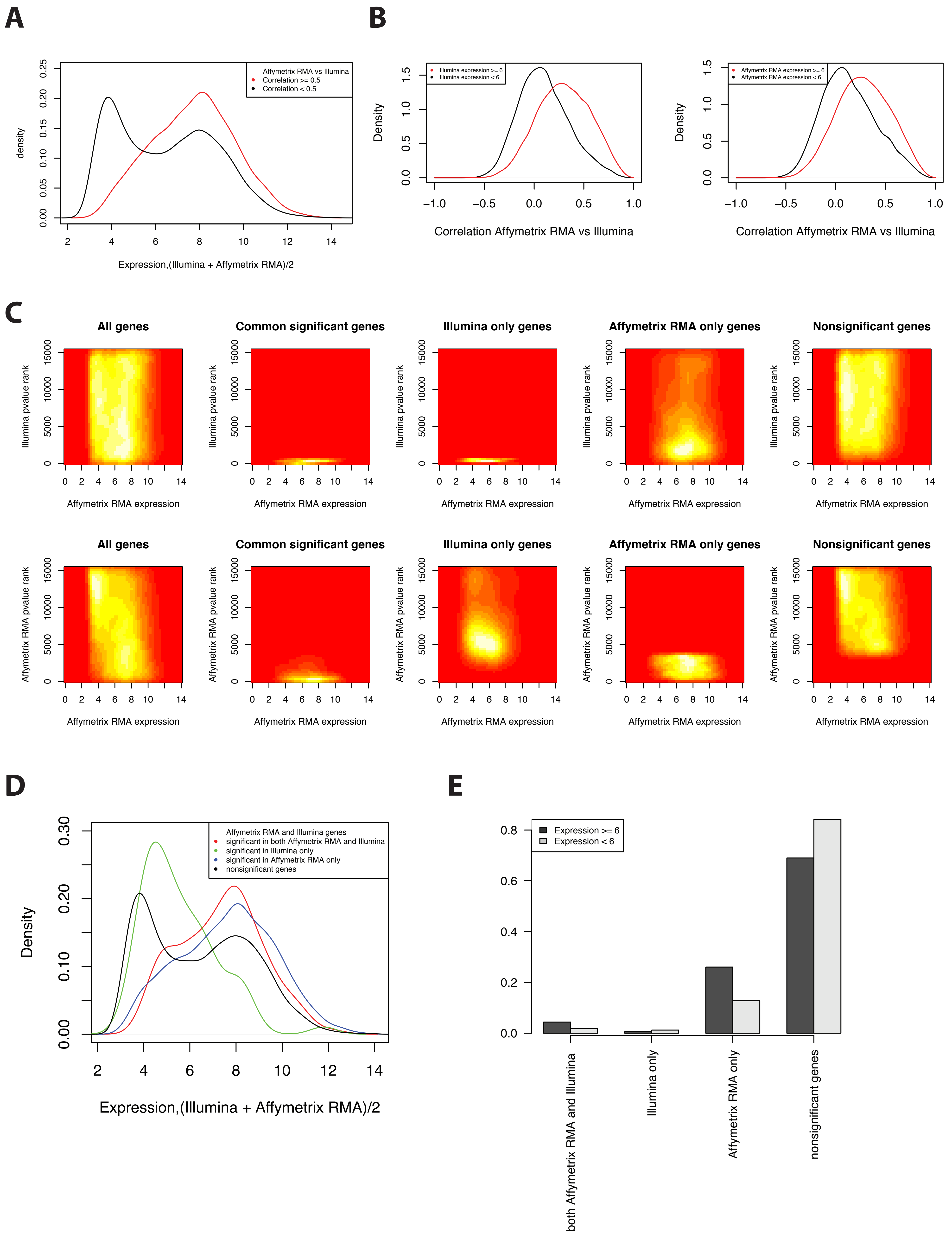

Additional File 1. Relationship between gene-wise correlation, significance group, and expression level. A) Density plot over mean Affymetrix and Illumina expression level for high- and low-correlation genes. While highly correlated genes have higher expression overall, genes with low correlation have a bimodal distribution with respect to expression level. The mean expression levels for high- and low-correlation genes are 7.703 and 6.467, respectively. The difference in mean expression between high- and low-correlation genes is significant ($p < 2.2 \times 10^{-16}$, Wilcoxon sum rank test). B) Density of correlation in high- and low-expression genes. Genes that are highly expressed have higher correlation. The mean correlation for genes with Illumina expression ≥ 6 and < 6 are 0.296 and 0.099, respectively ($p < 2.2 \times 10^{-16}$, t-test). The variances of the high and low Illumina expression genes are 0.070 and 0.063. The mean correlation for genes with Affymetrix expression ≥ 6 and < 6 are 0.279 and 0.136, respectively ($p < 2.2 \times 10^{-16}$, t-test). The variances of the high and low Affymetrix expression genes are 0.071 and 0.072. C) Two-dimensional density plots of Affymetrix expression value versus p-value ranks in different significance groups. D) Distribution of mean expression level for each significance group. G_{ai} and G_{ns} have distinct but broad and overlapping distributions. The mean expression levels for G_{ai}, G_i, G_a, and G_{ns} are 7.358, 5.579, 7.609, and 6.412, respectively. The variances for G_{ai}, G_i, G_a, and G_{ns} are 3.30, 2.59, 4.24, and 5.15, respectively. Using the t-test, G_{ai} is significantly different from G_i and G_a ($p < 2.2 \times 10^{-16}$ and = 0.005). Similarly, G_{ns} is significantly different from G_i and G_a ($p = 3.836 \times 10^{-8}$ and $< 2.2 \times 10^{-16}$). E) Distribution of high- and low-expression genes over the significance groups.
