# Supplementary material for: Platform dependence of inference on gene-wise and gene-set involvement in human lung development
Source: BMC Bioinformatics. 2009 Jun 19;10:189. doi: 10.1186/1471-2105-10-189 (PMC2711081; doi:10.1186/1471-2105-10-189)
Supplement: Additional file 2 — Relationship between gene-wise correlation and probe distance between the best-matched Affymetrix and Illumina probes. A) Distribution of probe distance for high- and low-correlation genes. Genes that are highly correlated have greater probe overlap. The median probe distances for high- and low-correlation genes are -25 and -21, respectively (p < 2.2 × 10^-16^, t-test). B) Distribution over correlation for probes in which the best-matched Affymetrix and Illumina probes are perfectly matched, partially overlapped, and non-overlapping. Correlation increases as the degree of probe overlap increases. The mean correlation for the perfectly matched, partially overlapped, and non-overlapping probes are 0.251, 0.215, and 0.169, respectively. The variances are 0.078, 0.077, and 0.070, respectively. Using the t-test, p perfect match, partial overlap = 6.097 × 10^-10^, p perfect match, no overlap < 2.2 × 10^-16^, and p partial overlap, no overlap = 3.202 097 × 10-14. C) Distribution of probe distance for each significance group. The mean probe distance for Gai, Gi, Ga, and Gns are 109.1, 250.7, 219.5, and 175.4, respectively. The variances are 2.15 × 10^5^, 6.16 × 10^5^, 2.18 × 10^5^, and 4.14 × 10^5^, respectively. D) Distribution of best-matched probes that are perfectly matched, partially overlapped, and non-overlapping between Affymetrix and Illumina over significance group. Although the distributions over the different probe distances appear similar, the χ2 test between probe distance and significance group showed an association between the two factors with p = 0.006. [file 1471-2105-10-189-S2.pdf]

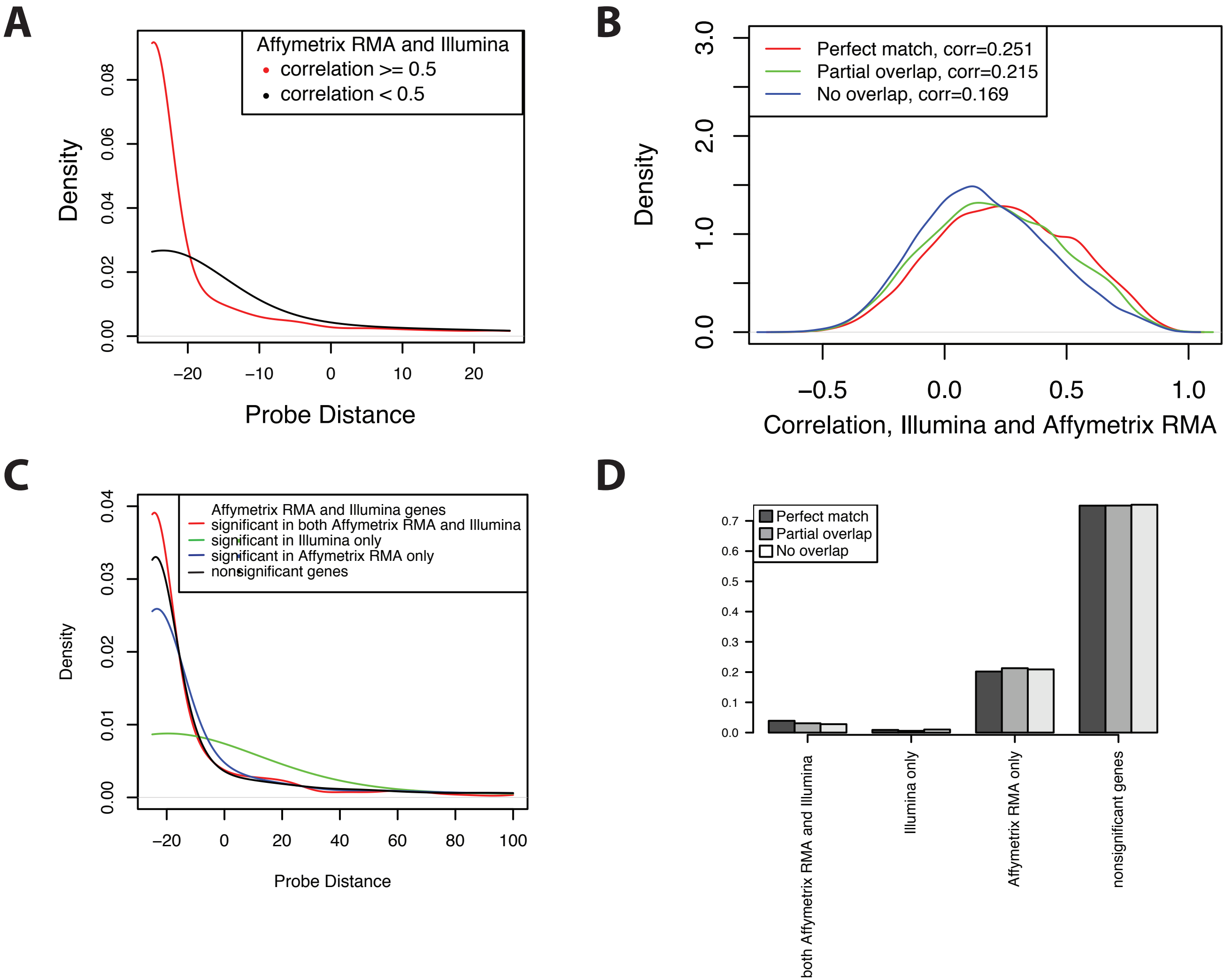

Additional File 2. Relationship between gene-wise correlation and probe distance between the best-matched Affymetrix and Illumina probes. A) Distribution of probe distance for high- and low-correlation genes. Genes that are highly correlated have greater probe overlap. The median probe distances for high- and low-correlation genes are -25 and -21, respectively ($p < 2.2 \times 10^{-16}$, t-test). B) Distribution over correlation for probes in which the best-matched Affymetrix and Illumina probes are perfectly matched, partially overlapped, and non-overlapping. Correlation increases as the degree of probe overlap increases. The mean correlation for the perfectly matched, partially overlapped, and non-overlapping probes are 0.251, 0.215, and 0.169, respectively. The variances are 0.078, 0.077, and 0.070, respectively. Using the t-test, $p_{\text{perfect match, partial overlap}} = 6.097 \times 10^{-10}$, $p_{\text{perfect match, no overlap}} < 2.2 \times 10^{-16}$, and $p_{\text{partial overlap, no overlap}} = 3.202097 \times 10^{-14}$. C) Distribution of probe distance for each significance group. The mean probe distance for G_{ai}, G_i, G_a, and G_{ns} are 109.1, 250.7, 219.5, and 175.4, respectively. The variances are 2.15×10^5, 6.16×10^5, 2.18×10^5, and 4.14×10^5, respectively. D) Distribution of best-matched probes that are perfectly matched, partially overlapped, and non-overlapping between Affymetrix and Illumina over significance group. Although the distributions over the different probe distances appear similar, the χ^2 test between probe distance and significance group showed an association between the two factors with $p = 0.006$.
