# Supplementary material for: Platform dependence of inference on gene-wise and gene-set involvement in human lung development
Source: BMC Bioinformatics. 2009 Jun 19;10:189. doi: 10.1186/1471-2105-10-189 (PMC2711081; doi:10.1186/1471-2105-10-189)
Supplement: Additional file 3 — Distribution over p-value rankings for high- and low-correlation genes. Highly correlated genes have lower p-value rankings with similar distributions in both Affymetrix and Illumina platforms. The mean Illumina p-value ranking for high- and low-correlation genes are 3956 and 8466 (p < 2.2 × 10^-16^, Wilcoxon sum rank test). The mean Affymetrix p-value ranking for high- and low-correlation genes are 4664 and 8315 (p < 2.2 × 10^-16^, Wilcoxon sum rank test). [file 1471-2105-10-189-S3.pdf]

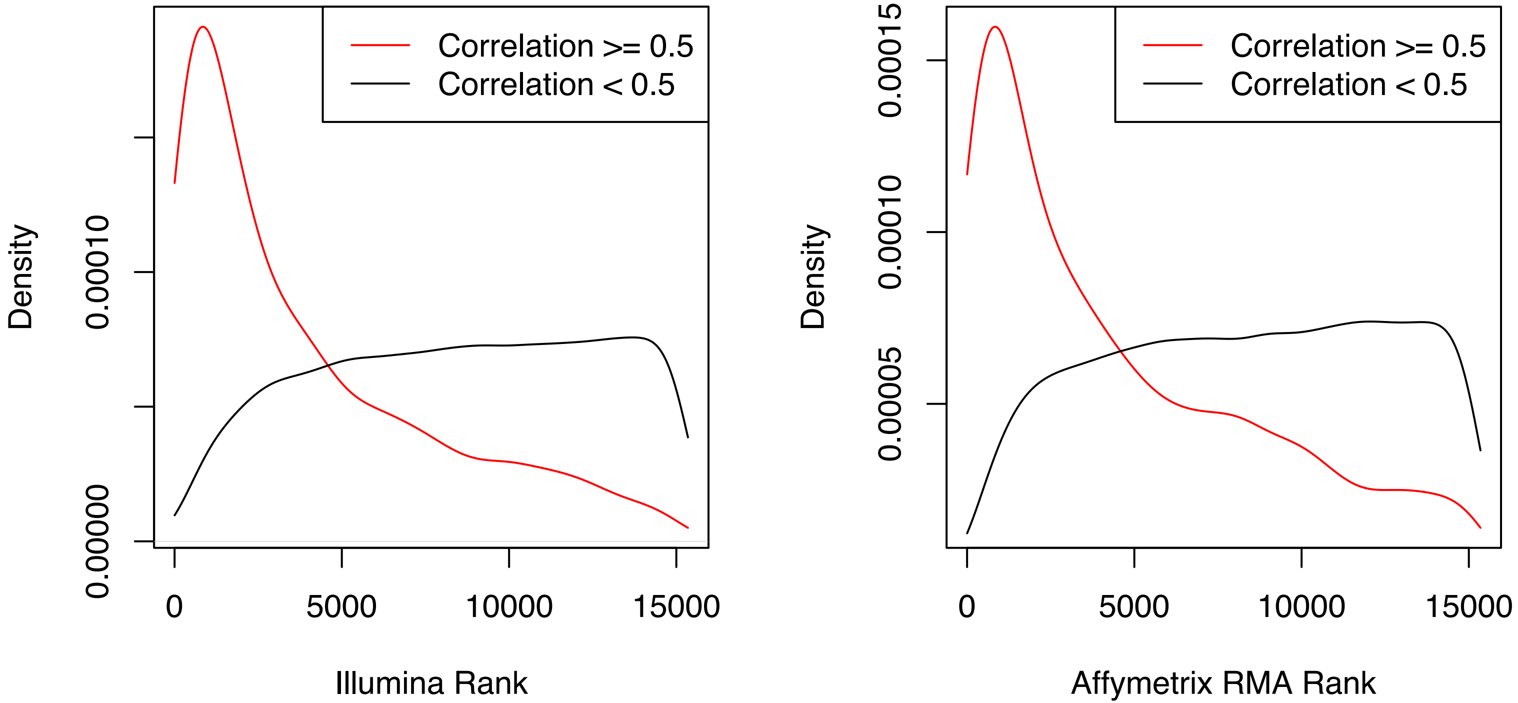

Additional File 3. Distribution over p-value rankings for high- and low-correlation genes. Highly correlated genes have lower p-value rankings with similar distributions in both Affymetrix and Illumina platforms. The mean Illumina p-value ranking for high- and low-correlation genes are 3956 and 8466 ($p < 2.2 \times 10^{-16}$, Wilcoxon sum rank test). The mean Affymetrix p-value ranking for high- and low-correlation genes are 4664 and 8315 ($p < 2.2 \times 10^{-16}$, Wilcoxon sum rank test).
