# Supplementary material for: Platform dependence of inference on gene-wise and gene-set involvement in human lung development
Source: BMC Bioinformatics. 2009 Jun 19;10:189. doi: 10.1186/1471-2105-10-189 (PMC2711081; doi:10.1186/1471-2105-10-189)
Supplement: Additional file 4 — Top 10 GO_slim ancestor terms. Comparison of GO_slim ancestor terms for Affymetrix and Illumina. [file 1471-2105-10-189-S4.doc]

**Additional File 4**. Top 10 GO_slim ancestor terms for Affymetrix and Illumina

| GO Class ID | Definition | % of significant GO categories with this ancestor | |
| --- | --- | --- | --- |
|  |  | Illumina | Affymetrix |
| GO:0008150 | Biological process | 17.99 | 18.73 |
| GO:0008152 | Metabolism | 6.85 | 5.99 |
| GO:0003674 | Molecular function | 6.85 | 6.93 |
| GO:0016043 | Cell organization and biogenesis | 5.78 | 4.68 |
| GO:0005575 | Cellular component | 5.78 | 5.62 |
| GO:0006139 | Nucleobase, nucleoside, nucleotide, and nucleic acid metabolism | 5.35 | 3.00 |
| GO:0005623 | Cell | 4.93 | 5.06 |
| GO:0005622 | Intracellular | 4.07 | 4.12 |
| GO:0006810 | Transport | 3.43 | 3.00 |
| GO:0003824 | Catalytic activity | 3.21 | 4.12 |
