# Supplementary material for: Platform dependence of inference on gene-wise and gene-set involvement in human lung development
Source: BMC Bioinformatics. 2009 Jun 19;10:189. doi: 10.1186/1471-2105-10-189 (PMC2711081; doi:10.1186/1471-2105-10-189)
Supplement: Additional file 5 — Significant KEGG pathways. Comparison of significant KEGG pathways between Affymetrix and Illumina. [file 1471-2105-10-189-S5.doc]

**Additional File 5.** Significant KEGG pathways

| **Illumina** | **Affymetrix RMA** |
| --- | --- |
| Cell cycle | Cell cycle |
| p53 signaling pathway | p53 signaling pathway |
| ECM-receptor interaction | ECM-receptor interaction |
| Small cell lung cancer | Small cell lung cancer |
| Selenoamino acid metabolism | Selenoamino acid metabolism |
| Antigen processing and presentation | Antigen processing and presentation |
| Glycosphingolipid biosynthesis - ganglioseries |  |
| Glyoxylate and dicarboxylate metabolism |  |
| One carbon pool by folate |  |
| Prion disease |  |
| Cholera - Infection |  |
| Bladder cancer |  |
|  | Methionine metabolism |
|  | Reductive carboxylate cycle (CO2 fixation) |
|  | Alkaloid biosynthesis II |
|  | Proteasome |
