# Supplementary material for: Platform dependence of inference on gene-wise and gene-set involvement in human lung development
Source: BMC Bioinformatics. 2009 Jun 19;10:189. doi: 10.1186/1471-2105-10-189 (PMC2711081; doi:10.1186/1471-2105-10-189)
Supplement: Additional file 6 — P-values of gene expressions in Affymetrix RMA, Illumina and p-values of ΔCt in quantitative PCR results. CCL20, CXCL3, and CXCL5 were not significant in both Affymetrix and Illumina. CD36, SFTPB, SFTPC, and TUBB2B were significant in both Affymetrix and Illumina. Genes that were significant in both platforms were also significant in qPCR. [file 1471-2105-10-189-S6.pdf]

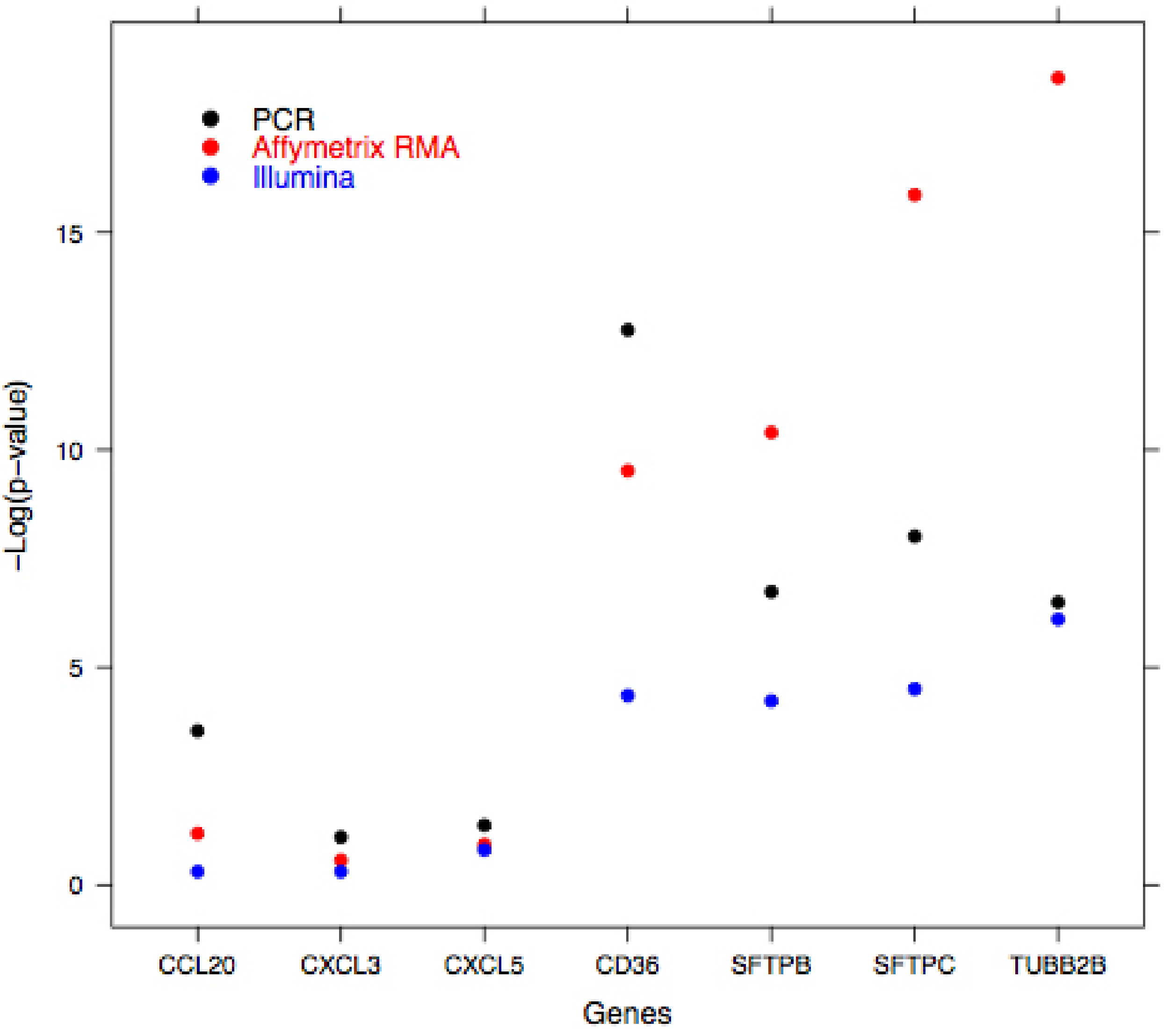


**Additional File 6.** P-values of gene expressions in Affymetrix RMA, Illumina and p-values of ΔCt in quantitative PCR results. CCL20, CXCL3, and CXCL5 were not significant in both Affymetrix and Illumina. CD36, SFTPB, SFTPC, and TUBB2B were significant in both Affymetrix and Illumina. Genes that were significant in both platforms were also significant in qPCR.
