# Supplementary material for: Platform dependence of inference on gene-wise and gene-set involvement in human lung development
Source: BMC Bioinformatics. 2009 Jun 19;10:189. doi: 10.1186/1471-2105-10-189 (PMC2711081; doi:10.1186/1471-2105-10-189)
Supplement: Additional file 7 — Local statistics of genes in a significant KEGG pathway. Empirical distribution function of the ranked local statistics of genes in a significant KEGG pathway (KEGG:04110) against that of all genes (A = Affymetrix, B = Illumina). [file 1471-2105-10-189-S7.pdf]

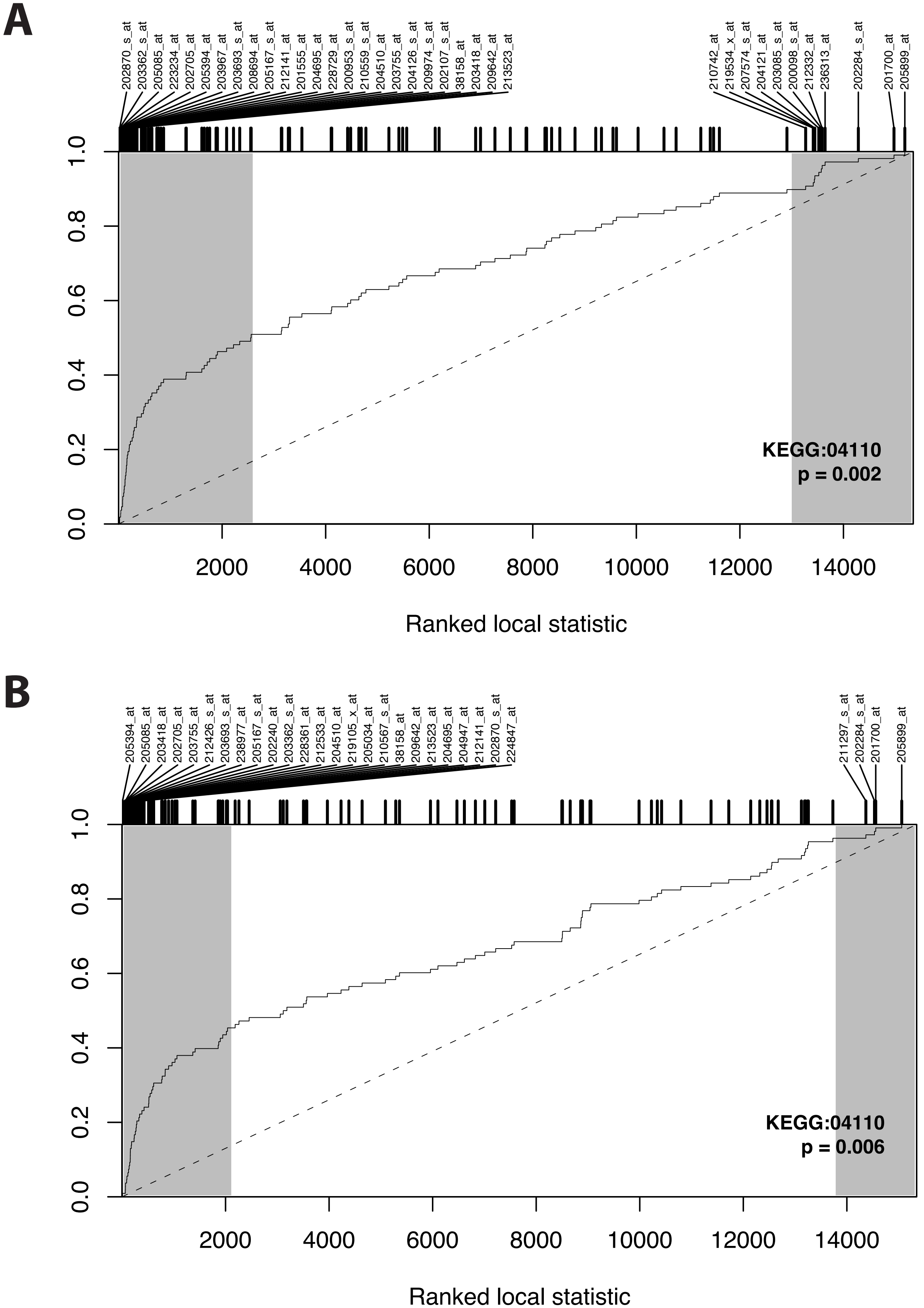

Additional File 7. Empirical distribution function of the ranked local statistics of genes in a significant KEGG pathway (KEGG:04110) against that of all genes (A = Affymetrix, B = Illumina).
