# Supplementary material for: Platform dependence of inference on gene-wise and gene-set involvement in human lung development
Source: BMC Bioinformatics. 2009 Jun 19;10:189. doi: 10.1186/1471-2105-10-189 (PMC2711081; doi:10.1186/1471-2105-10-189)
Supplement: Additional file 8 — Piecewise constant model for gene expression. Affymetrix gene expression fitted to a piecewise constant model for two genes. [file 1471-2105-10-189-S8.pdf]

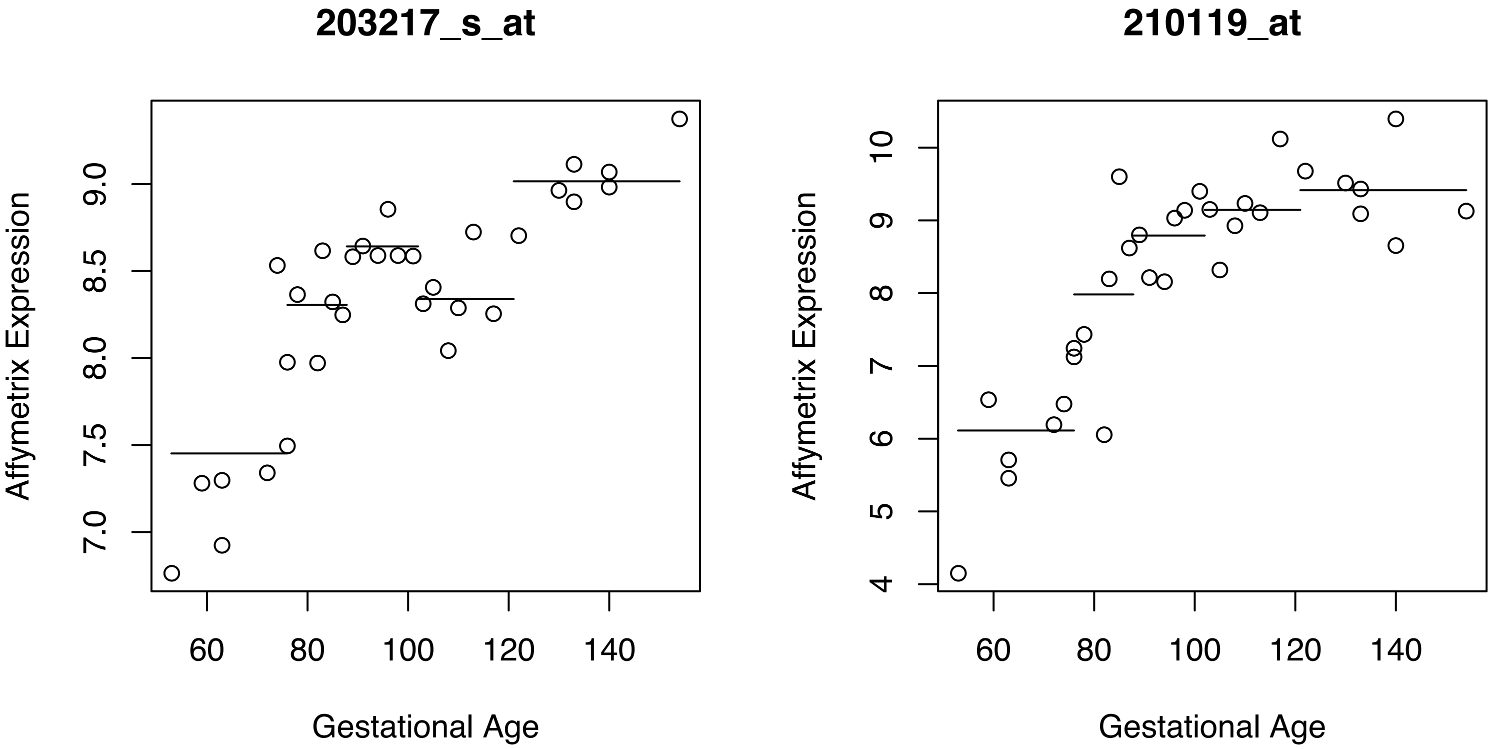

Additional File 8. Affymetrix gene expression fitted to a piecewise constant model for two genes.
